# Universal mental health training: policy proposal for national rollout of the new scalable intervention in Ukraine

**DOI:** 10.12688/openreseurope.20116.2

**Published:** 2026-01-13

**Authors:** Viktoriia Gorbunova, Vitalii Klymchuk

**Affiliations:** 1 Department of Behavioural and Cognitive Sciences, University of Luxembourg, Esch-sur-Alzette, Luxembourg District, L-4365, Luxembourg; 2 Department of Applied and Social Psychology, Zhytomyr Ivan Franko State University, Zhytomyr, Zhytomyrs'ka oblast, 10008, Ukraine; 3 Department of Social Sciences, University of Luxembourg, Esch-sur-Alzette, Luxembourg District, L-4365, Luxembourg

**Keywords:** mental health, universal mental health training, frontline professionals, public health

## Abstract

This policy proposal outlines the challenges and relevant policy actions for scaling up Universal Mental Health Training (UMHT) in Ukraine. The UMHT is an educational program that aligns with Ukraine’s new Law on the Mental Health Care System adopted in 2025, and is supported by data from pilot research. It equips frontline professionals - those who work closely with clients and service users, including teachers, police officers, and social workers - with foundational skills to identify mental health concerns, provide initial support, and facilitate referrals to specialized services. The UMHT implementation has the potential to bridge the mental health treatment gap by creating a broad base of accessible community-level helpers.

Key policy challenges include integrating mental health support into existing professional roles, ensuring sustainable financing beyond international grants, maintaining quality through ongoing supervision, and countering stigmas. To address this, the proposal advocates embedding the UMHT competencies in occupational standards, leveraging intersectoral coordination through the Coordination Center for Mental Health, shifting towards local budget allocations and academic partnerships, and pursuing continuous professional development.

## Introduction

In recent years, mental health has moved to the forefront of Ukraine’s national agenda. The cumulative stress from social and political instability, the COVID-19 pandemic, the Russian invasion of Eastern Ukraine in 2014, and the full-scale invasion in 2022 has created an unprecedented need for psychological support across the population. According to WHO, 72% of adults in Ukraine have experienced mental health difficulties over the past year ( WHO, 2025), 36% of Ukrainian adults have symptoms of at least one mental disorder ( Martsenkovskyi *et al.*, 2024), and the prevalence of post-traumatic stress in adults ranges from 15% to 41% ( Yasenok *et al.*, 2025).

In response, Ukraine’s government, supported by a professional society, launched three consecutive initiatives. First, adopting the Concept Note on Mental Health Care in Ukraine up to 2030 ( Cabinet of Ministers of Ukraine (№ 1018-р), 2017) in 2017. This was the first-ever official document, proclaiming the transformation of the Ukrainian mental health system into a community-based and human-rights-based system. Second, the Action Plan for 2021–2023 ( Cabinet of Ministers of Ukraine (№ 1215-р), 2021) in 2021 and its prolongation ( Cabinet of Ministers of Ukraine, 2024) for 2024–2026 to support the Concept Note. It created the actionable directives for national and regional stakeholders on the integration of mental health into primary healthcare and developing community-based mental health services. Third, in 2025, a new law on the Mental Health Care System in Ukraine ( Verkhovna Rada of Ukraine, 2025) was adopted. This document created a ground for the integration of mental health in all policies, making it everyone's business. Mentioned legislative endorsements reconceptualise mental health care in Ukraine as a broad, community-based system in contrast to the old one, restricted to psychiatric institutions. The ongoing challenge is to keep turning these high-level commitments into practical support that reaches people in their daily lives. This is where the idea of Universal Mental Health Training (UMHT) comes in – an initiative to train and mobilise frontline professionals (such as teachers, police officers, non-clinical social workers, and others) as a first line of community mental health support.

This essay provides an overview of the UMHT initiative and discusses the key policy challenges and solutions for its national rollout by 2030. Drawing on recent pilot effectiveness and feasibility studies ( Gorbunova *et al.*, 2024; Gorbunova *et al.*, 2025) and aligning with Ukraine’s policy framework, it envisions how the UMHT can sustainably strengthen the country’s mental healthcare system. The discussion is relevant not only to policymakers and academics but also to practitioners and community stakeholders interested in building a more resilient society.

## Universal Mental Health Training: background and pilot implementation results

Universal Mental Health Training, developed in Ukraine in 2021, is an educational program for frontline professionals that aims to bridge the mental health treatment gap (a disparity between the number of people who need mental health care and those who actually receive it). Reflecting on the WHO-endorsed task-shifting approach as a partial delegation of some mental health support tasks to trained non-mental health service providers ( Javadi *et al.*, 2017), the UMHT equips frontline workers with basic skills to recognize when someone is experiencing a mental health issue, provides initial comfort and advice, and guides them towards professional help if needed ( Gorbunova *et al.*, 2024). In essence, it turns a wide range of public-facing professionals into “gatekeepers” or community guardians of mental well-being. In this way, UMHT supports multi-sectoral public health, treating mental health not only as a healthcare issue but also as a shared social responsibility – in line with the new Ukrainian Law on Mental Health Care System, reframing of mental health as a “socially significant field” ( Verkhovna Rada of Ukraine, 2025).

For example, a school teacher trained under the UMHT can identify a student who requires mental health support and talk with them and their family, providing assistance and guidance towards professional help if necessary. Another case is that of a police officer who can de-escalate a situation with a distressed individual and link them to services. This approach draws on global best practices such as the WHO’s Mental Health Gap Action Programme ( WHO, 2008) and Mental Health First Aid ( Kitchener & Jorm, 2008), tailoring them to Ukraine’s context. Detailed information on PFA and MHFA in this context is provided in our previous publication ( Gorbunova *et al.*, 2025).

The policy recommendations presented in this article are grounded in a multi-year pilot phase of the Universal Mental Health Training (UMHT) program conducted in Ukraine between 2021 and 2024 ( Gorbunova *et al.*, 2024; Gorbunova *et al.*, 2025). We utilised a mixed-methods approach to assess the feasibility and effectiveness of a "task-shifting" model designed for non-mental health professionals. The pilot involved two primary studies: a feasibility analysis (n=714) and a controlled trial (n=307). The training targeted frontline professionals (FLPs) who are often the first point of contact for individuals in distress, including police officers, educators, social workers, and emergency responders. Between 2021 and early 2024, the program scaled significantly, delivering 152 training events and reaching over 3,000 individuals.

The training efficacy was measured using standardised pre- and post-test assessments and Likert-scale surveys focusing on knowledge, readiness, and usability.

- **Knowledge and Readiness:** Participants demonstrated a statistically significant increase in mental health knowledge scores (p < 0.05). Furthermore, their self-reported readiness to interact with individuals experiencing mental health issues rose from a baseline mean of 3.28 to 4.46 (SD=0.65) on a 5-point scale.
- **Feasibility and Acceptability:** The UMHT model achieved high acceptability ratings from both trainers (4.81/5) and trainees (4.78/5).
- **Systemic Usability:** Across different sectors, the training showed consistent usability scores, with police (0.67) and educators (0.68) reporting high confidence in applying the five-step "Recognise, Validate, Support, Refer, and Ensure" model in their daily duties.

Beyond quantitative metrics, the pilot phase revealed a shift in organizational culture, particularly within law enforcement and education. Qualitative feedback suggested that the training helped destigmatize mental health challenges in high-stress work environments and provided a clear, actionable framework for "work-site crises." These empirical results confirm that the UMHT model is not only acceptable to the target audience but also a highly usable tool for strengthening community-based mental health support systems.

UMHT dissemination was organised using the Training of Trainers (ToT) approach, which involves preparing trainers (selected among mental health professionals), followed by their accreditation and entry into the UMHT trainer register. The trainers then trained and supervised the frontline professionals ( Gorbunova *et al.*, 2024). Demand for training has grown rapidly each year, indicating its feasibility. In 2023 alone, 90 training events were conducted, increasing from 27 in 2021 ( Gorbunova *et al.*, 2025). These numbers suggest a strong foundation for rolling out the UMHT as a national program, aiming to make mental health support in Ukraine truly universal – available in a village, town, school, and service desk wherever it might be needed.

## Embedding Equity and Inclusion

The "Universal" in UMHT signifies a commitment to reaching all segments of the Ukrainian population, particularly those historically underserved or disproportionately affected by the conflict. Equity is embedded into the design and rollout of the initiative through three strategic pillars: targeted outreach, cultural adaptation, and disaggregated monitoring.

### Reaching Marginalised and Rural Populations

To address the "treatment gap" in rural areas, where specialised mental health services are scarce, the UMHT prioritises training for local frontline professionals (e.g., village council members, postal workers, and local primary care feldshers, etc.). For internally displaced persons (IDPs), training can be delivered explicitly within collective centres and humanitarian hubs, ensuring that those in temporary housing have access to professionals equipped with trauma-informed support skills.

### Cultural Adaptation

While the program is standardised, the delivery is adapted to the socio-cultural nuances of specific groups. Modules include specific scenarios involving veterans, persons with disabilities, and ethnic minorities (e.g., the Roma community), ensuring that frontline professionals can recognise and support distress through a culturally sensitive lens.

### Data-Driven Equity Monitoring

To avoid "blind spots" in the national rollout, the M&E framework must include the collection of disaggregated data. This will allow for the tracking of training penetration and effectiveness across different demographics, ensuring that resources are allocated to regions or groups with the highest need.

## Challenges in the UMHT scaling up at the national level

Scaling up UMHT to the national level by 2030 is an ambitious goal that faces **several challenges**.

*Integrating into Existing Systems.* One major challenge is integrating mental health support into the daily workflow of various frontline professionals without causing disruptions. Frontline workers, such as nurses, teachers, and police officers, are already busy with their primary duties. Asking them to take on additional mental health support tasks can be seen as a burden or distraction. There might be resistance or simply a lack of time and energy to apply the trained skills unless using them is beneficial for primary duties and well integrated into the work schedule. Integration will require adjusting job descriptions, securing management support, and demonstrating that attending to mental health helps and eases the achievement of core goals (such as a teacher’s goal of better student performance or a nurse’s goal of holistic patient health).

*Funding and Sustainability.* Initially, much of the pilot stage training was supported by grants and international aid. However, reliance on external funding is not sustainable for national programmes. The challenge is to obtain stable funding in the long term, given the many competing priorities in a country during war and rebuilding. The program’s clear benefits and cost-effectiveness can win political and financial support, whereas integrating UMHT into university curricula and engaging higher education institutions and continuing professional education providers in the program’s dissemination will ease additional spending. 

*Workforce Quality Assurance.* Although the UMHT relies on existing frontline workers, ensuring quality control across a broad rollout remains a challenge. Maintaining trainees’ engagement and updating their skills over time requires ongoing supervision and upskilling, which in turn requires a substantial increase in trained professionals and the integration of UMHT into the system of continuous professional development across different sectors (which are usually non-integrated).

*Public and Professional Attitudes.* Some frontline professionals may initially doubt the value of mental health training, seeing it as outside and excessive (“Why should a fireman need to know about depression?”). Likewise, members of the public might be sceptical or reluctant to accept help from a non-professional in the field, especially given the stigma surrounding mental health. Both issues are related to stigma reduction, and raising awareness may help overcome attitudinal barriers.

## Policy actions to overcome the challenges and scale up the UMHT

**Figure 1.**
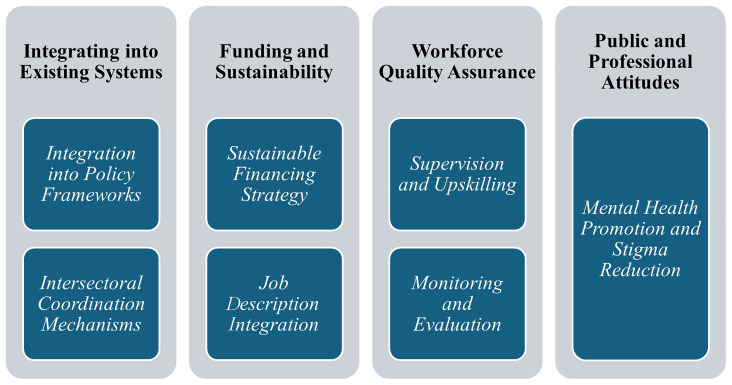
Challenges and policy-related solutions for UMHT implementation in Ukraine.

As briefly mentioned alongside the challenges, there are solutions for each of them. Some are already embedded in the program, piloted, and enhanced during the pilot implementation. Some were in the field of **policy actions** ( Figure 1).

– *Integration into Policy Frameworks.* One solution is to anchor the UMHT in national policy. Ukraine’s law on the Mental Health Care System already provides an umbrella mandate for community-based and intersectoral approaches, stating them as the central principles of state mental health policy ( Verkhovna Rada of Ukraine, 2025). Building on this, the government can issue specific directives or action plans dedicated to supporting UMHT. For example, a government order could require all relevant ministries to include UMHT training targets in their annual plan. In addition, including basic mental health support in the occupational standards and UMHT itself in the educational or continuous professional education curricula for different frontline professionals will make it part of the system. When frontline workers see that mental health support is officially part of their job expectations (backed by orders and directions), they are more likely to accept it as a natural part of their job.
– *Intersectoral Coordination Mechanisms*. Practical coordination across sectors can be achieved through bodies such as the Coordination Center for Mental Health (CCMH), which the Cabinet of Ministers of Ukraine purposely established to coordinate efforts and facilitate collaboration among ministries, institutions, organizations, local government bodies, and the non-governmental sector ( Cabinet of Ministers of Ukraine, 2023). For instance, CCMH can hold a UMHT trainer register and work with different ministries through liaison persons to support and control the performance of the UMHT action plan.
– *Sustainable Financing Strategy.* In terms of funding, it is crucial to switch from external aid to domestic funding over the long term. The UMHT is an educational capacity-building initiative that allows its financial integration into higher and Continuous Professional Development (CPD). The preparation of UMHT trainers can occur during higher education training for psychologists or psychiatrists, with obligatory fulfilment of all training requirements. The existing pool of UMHT trainers and newly prepared trainers can be engaged in CPD of different types of frontline professionals.
– *Job description integration.* At the local level, it is important to integrate the provision of basic mental health support into documents that regulate the job functions of each professional (in most cases, job description). Job descriptions in unregulated professions are developed locally by each organization itself, so it is possible to implement such changes quickly after the personnel go through UMHT. For the regulated professions that have occupational standards ( Cabinet of Ministers of Ukraine (№ 373), 2017), such changes have to start with the amendments in these standards, outlining the UMHT-based list of competencies, among others.
– *Supervision and Upskilling*. It is important to establish permanent supervision or an on-site mentoring process with repetitive upskilling sessions to ensure that the workforce’s capacity to maintain mental health support skills is updated long after passing the initial UMHT. They can be embedded into the work routine as part of a workplace-installed CPD system, which is regulated by the relevant ministerial orders in healthcare, education, social work, etc. ( Cabinet of Ministers of Ukraine, 2019; Cabinet of Ministers of Ukraine (№ 725), 2021; Ministry of Social Policy of Ukraine, 2014).
– *Monitoring and Evaluation (M&E)* are essential for ensuring the quality, accessibility, and impact of UMHT. A robust M&E framework must be created, enabling continuous assessment of training and support delivery, identifying gaps and areas for improvement, while ensuring that interventions align with evidence-based practices and meet the needs of diverse populations. Additionally, M&E s play a crucial role in assessing equity in mental health support, ensuring that vulnerable and underserved groups receive adequate support.
– *Mental Health Promotion and Stigma Reduction.* To increase the buy-in of the UMHT by relevant local and national stakeholders, it is important to continue existing national and local mental health promotion and stigma reduction campaigns (How Are You? ^1^, Mental Health Ambassador Program ^2^. There Is More ^3^) and integrate into them the idea that mental health is everyone's business. Embedding messages about the role and importance of the comprehensive multidisciplinary approach and the possibility of each professional providing basic mental health support to people in need can increase support for the UMHT and ease its integration into existing services. The frontline workers themselves may actually find their work easier in some respects – many report that after UMHT training, they felt better prepared when encountering mental health issues. Empowerment can lead to greater job satisfaction and greater effectiveness, and less stigma.

## Monitoring and evaluation framework for national scaling

To ensure the quality, sustainability, and impact of the UMHT national rollout, a comprehensive Monitoring and Evaluation (M&E) framework must be implemented. This framework proposal transitions from measuring immediate training outputs to evaluating long-term systemic changes in frontline professional (FLP) behaviour and referral efficacy.

**1. Quality Assurance and Workforce Retention** The quality of training delivery can be maintained through a standardised "Training of Trainers" (ToT) certification process, followed by periodic "Supervision of Supervision" sessions. To address the concern of skill decay, the program can implement longitudinal follow-ups at 6 and 12 months post-training, utilising self-assessment scales and vignette-based testing to measure the retention of the "5-step interaction model."

**2. Impact on Referral Pathways** A key indicator of success is the "closing of the referral loop." The M&E plan can utilise anonymised referral tracking logs to determine if referrals made by FLPs (e.g., police or teachers) successfully lead to intake at specialised mental health services. This can be cross-referenced with "referral quality" audits (assessing whether FLPs are providing the necessary information to people and mental health professionals to ensure a seamless transition of care).

**3. M&E Schematic Framework** The following table outlines the key indicators and data sources identified for the national rollout ( Table 1)

**Table 1.** M&E Domains and KPI.

Evaluation Domain	Key Performance Indicator (KPI)	Data Source / Tool
**Training Quality**	% of trainers achieving >85% on fidelity checklists during delivery.	Observation checklists & Participant satisfaction surveys.
**Skill Retention**	Mean change in "Readiness to Interact" scores at 6-month follow-up.	Longitudinal digital surveys (Likert-scale).
**Behavioral Impact**	Frequency of use of the 5-step model in daily professional duties.	Workplace "Usage Diaries" or Quarterly professional audits.
**Referral Pathway**	% increase in successful "warm handovers" to mental health specialists.	Integrated Referral Tracking System or Service intake logs.
**Systemic Change**	Documented reduction in mental health stigma within participating institutions.	Culturally adapted tools for measuring stigma

## Looking ahead to 2030 – Expected outcomes

If the UMHT initiative is implemented with these solutions in mind, Ukraine’s mental health landscape will appear markedly different by 2030. Based on the data from the feasibility study and pilot trial ( Gorbunova *et al.*, 2024; Gorbunova *et al.*, 2025), We can expect to see a much larger proportion of the population receiving help for mental health issues early, rather than waiting until crises require hospitalisation. Every school, clinic, workplace, and community centre would have someone equipped to recognise mental health conditions, initiate and lead the conversation on the matter, give first support, refer to professional help, and ensure that help is received. The mental health gap – those who need care versus those who receive it – should shrink, reflecting increased accessibility. The strain on specialised psychiatric services may ease, as mild and moderate cases are managed or referred to appropriately at the community level. Importantly, mental health will be normalised as a common aspect of public services, echoing the law’s intent to treat it as a normal part of overall health and social well-being. It is important to note that such changes would occur without substantive increases in the number of mental health workers, and therefore with minimal financial investment.

## Conclusion

Ukraine is a key moment in shaping a community-based mental health system. The UMHT initiative is a way to create a system of mental health community helpers and meet the population's mental health needs. While challenges in policy coordination, funding, workforce, and societal attitudes exist, they can be overcome through careful planning, strong political will, and inclusive stakeholder engagement.

By adopting the outlined policy actions – from integrating UMHT into the fabric of multiple sectors–to ensure sustainable support and continuous quality improvement, Ukraine can successfully roll out the UMHT by 2030. A teacher’s comforting words, a police officer’s understanding response, and a pharmacist’s gentle referral – these small acts, multiplied across the country, will form a powerful mental health support network. By 2030, as Ukraine continues to recover and develop, the UMHT could enhance a mentally healthier and more connected population empowered by mutual support.

Given the rapidly rising prevalence of mental health conditions across Ukraine, the urgency of implementing scalable mental health solutions becomes even more evident, underscoring the public health imperative at a population level.

## Ethics and consent

Ethical approval and consent were not required

## Data Availability

No new data were created or analyzed in this study. Therefore, data sharing is not applicable to this article.

## Footnotes

^1^ https://howareu.com/

^2^ https://www.mh4u.in.ua/ambasador/

^3^ https://www.mh4u.in.ua/dali-ye/
