# Supplementary material for: Universal mental health training: policy proposal for national rollout of the new scalable intervention in Ukraine
Source: Open Res Eur. 2026 Jan 13;5:119. Originally published 2025 May 6. [Version 2] doi: 10.12688/openreseurope.20116.2 (PMC12906641; doi:10.12688/openreseurope.20116.2)
Supplement: Peer review history

# Reviewer report: Reviewer response for version 2

**DOI:** 10.21956/openreseurope.24471.r68020

**Published:** 2026-02-16

**Authors:** Ling Wang

**Affiliations:** 1 King's College London, London, UK

happy to endorse indexing.

Is the argument persuasive and supported by appropriate evidence?

Yes

Is the work clearly and cogently presented?

Yes

Is the topic of the essay discussed accurately in the context of the current literature?

Yes

Does the essay contribute to the cultural, historical, social understanding of the field?

Yes

Reviewer Expertise:

mental health and psychosocial support during and beyond emergencies to routine services

I confirm that I have read this submission and believe that I have an appropriate level of expertise to confirm that it is of an acceptable scientific standard.

# Reviewer report: Reviewer response for version 2

**DOI:** 10.21956/openreseurope.24471.r68019

**Published:** 2026-02-14

**Authors:** Kathryn E Kanzler, Chase Aycock

**Affiliations:** 1 Department of Medicine; Department of Psychiatry & Behavioral Sciences, Baylor College of Medicine, Houston, Texas, USA; 2 Joint Base San Antonio - Wilford Hall Ambulatory Surgical Center, Lackland AFB, Texas, USA

We appreciate the authors’ responsiveness to feedback from all reviewers. This essay makes an even stronger case now for the critical need to disseminate Universal Mental Health Training (UMHT) in Ukraine. We hope the urgent call to action is heeded, as scaled-up UMHT has potential to impact not just Ukraine, but to serve as an international public health model, demonstrating how people can care for one another across sectors, thereby bolstering the mental health of entire populations. Thank you again for inviting us to participate as reviewers.

Is the argument persuasive and supported by appropriate evidence?

Yes

Is the work clearly and cogently presented?

Yes

Is the topic of the essay discussed accurately in the context of the current literature?

Partly

Does the essay contribute to the cultural, historical, social understanding of the field?

Yes

Reviewer Expertise:

task-shifting; implementation science; health equity; chronic disease management

We confirm that we have read this submission and believe that we have an appropriate level of expertise to confirm that it is of an acceptable scientific standard.

# Reviewer report: Reviewer response for version 1

**DOI:** 10.21956/openreseurope.21760.r58203

**Published:** 2025-09-23

**Authors:** Ling Wang

**Affiliations:** 1 King's College London, London, UK

This article presents a timely and thoughtful policy proposal centered on the national scaling of Universal Mental Health Training (UMHT) in Ukraine. In light of the ongoing humanitarian crisis and the country’s recent legislative reforms, the authors rightly argue that there is a critical opportunity to reimagine Ukraine’s mental health system through more community-based and preventive approaches.

 The proposal outlines how frontline professionals such as teachers, police officers, and social workers can be trained to recognize signs of mental distress, offer basic support, and refer individuals to more specialized care when necessary. The initiative is commendably positioned within Ukraine’s broader health policy landscape, particularly in relation to the 2025 Law on the Mental Health Care System. It draws inspiration from global task-sharing models, such as the WHO’s mhGAP and Mental Health First Aid programmes.

 The strengths of the paper lie in its practical orientation and policy relevance. It addresses genuine systemic challenges—particularly around integration, funding, workforce quality, and stigma—and offers a set of grounded, actionable recommendations to overcome them. The discussion around embedding mental health competencies into occupational standards, coordinating across sectors through a central body, and shifting from donor reliance to sustainable domestic funding is especially well argued. These proposals demonstrate a systems-level understanding of how such a training initiative might take root in the real-world complexities of Ukrainian governance and service delivery.

 That said, there are several areas where the manuscript would benefit from further strengthening. The most immediate concern is the limited presentation of data from the UMHT pilot studies. While the article references pilot work conducted between 2021 and 2024, it offers very little detail about the design, sample, methodology, or concrete findings. It would significantly improve the credibility of the proposal if the authors could summarize key aspects of this pilot phase—for example, how many individuals were trained, what kinds of professionals were involved, what evaluation tools were used, and what changes were observed in knowledge, confidence, or behaviour. Even a brief table or narrative summary would suffice. At present, the lack of empirical detail undermines the strength of the policy recommendations that follow.

 A second point relates to the conceptual positioning of UMHT. Although the article references WHO-endorsed approaches and touches on Mental Health First Aid, it misses the opportunity to explicitly situate UMHT in relation to Psychological First Aid (PFA), a widely recognized framework for providing initial psychosocial support in crisis settings. Given the similarities in ethos and content, a more direct comparison or clarification would be useful—not only to highlight what UMHT borrows or adapts from PFA, but also to establish what makes it distinctively Ukrainian. This would help readers, particularly international policymakers and practitioners, to better understand the model’s grounding in evidence-based practice.

 Furthermore, while the article rightly acknowledges the importance of supervision, quality assurance, and ongoing professional development, the discussion around monitoring and evaluation remains rather vague. A national rollout of this scale would benefit from a clear framework for assessing training quality, workforce retention of skills, and impact on referral pathways. The authors might consider outlining a basic monitoring and evaluation plan, even if in schematic form, identifying key indicators and potential data sources. This would give the proposal greater rigour and support future scalability and adaptation.

 Equity is another area that warrants further attention. While the UMHT initiative is positioned as universal, there is little discussion of how it will reach or serve marginalized populations—such as rural residents, displaced persons, or minority groups. Given Ukraine’s socio-political context and the disparities in mental health access, the authors should consider briefly reflecting on how equity considerations will be embedded in the design, delivery, and evaluation of UMHT. This could include measures such as targeted outreach, linguistic or cultural adaptation, or disaggregated monitoring data.

 Finally, a few stylistic and language issues are worth noting. The manuscript would benefit from minor editorial refinement, particularly in terms of phrasing and grammatical consistency. Terms like “stigma redaction” should be corrected to “stigma reduction”, and long paragraphs could be broken down for improved readability. Additionally, Figure 1, while helpful, could use a clearer caption and more legible formatting to ensure accessibility across platforms.

 In conclusion, this policy paper makes a valuable and forward-thinking contribution to the mental health policy discourse in Ukraine and beyond. It successfully frames mental health as a shared societal responsibility and offers practical routes to embedding support mechanisms within the existing workforce. However, in order to be scientifically sound and persuasive for both policymakers and academic readers, the authors could strengthen the evidence base, clarify conceptual linkages to global models like PFA, and more explicitly outline strategies for evaluation and equity. These revisions are not overly burdensome, but they are essential for making the proposal as impactful and actionable as it aims to be.

 Major revision required.

Is the argument persuasive and supported by appropriate evidence?

Yes

Is the work clearly and cogently presented?

Yes

Is the topic of the essay discussed accurately in the context of the current literature?

Yes

Does the essay contribute to the cultural, historical, social understanding of the field?

Yes

Reviewer Expertise:

mental health and psychosocial support during and beyond emergencies to routine services

I confirm that I have read this submission and believe that I have an appropriate level of expertise to confirm that it is of an acceptable scientific standard, however I have significant reservations, as outlined above.

# Response

**Published:** 2025-12-26

**Authors:** Viktoriia Gorbunova

**Affiliations:** Department of Behavioural and Cognitive Sciences, University of Luxembourg, Esch-sur-Alzette, Luxembourg District, Luxembourg

We would like to thank the reviewer for their thoughtful and constructive feedback on our manuscript. Their insights have been invaluable in strengthening the empirical foundation and practical framework of this policy proposal. In response to the reviewer's comments, we have significantly revised the manuscript, addressing all the issues.  

**Comment**: The most immediate concern is the limited presentation of data from the UMHT pilot studies. While the article references pilot work conducted between 2021 and 2024, it offers very little detail about the design, sample, methodology, or concrete findings. It would significantly improve the credibility of the proposal if the authors could summarize key aspects of this pilot phase—for example, how many individuals were trained, what kinds of professionals were involved, what evaluation tools were used, and what changes were observed in knowledge, confidence, or behaviour. Even a brief table or narrative summary would suffice. At present, the lack of empirical detail undermines the strength of the policy recommendations that follow. 

**Response**: We have added a narrative summary, detailing the pilot phase conducted between 2021 and 2024. This includes data from our controlled trial (n=307) and feasibility study (n=714), showing a significant increase in mental health knowledge (p < 0.05) and a high usability score among police (0.67) and educators (0.68). We have clarified that over 3,000 professionals across Ukraine have been trained to date, providing a robust empirical foundation for our policy recommendations.

**Comment**: A second point relates to the conceptual positioning of UMHT. Although the article references WHO-endorsed approaches and touches on Mental Health First Aid, it misses the opportunity to explicitly situate UMHT in relation to Psychological First Aid (PFA), a widely recognized framework for providing initial psychosocial support in crisis settings. Given the similarities in ethos and content, a more direct comparison or clarification would be useful—not only to highlight what UMHT borrows or adapts from PFA, but also to establish what makes it distinctively Ukrainian. This would help readers, particularly international policymakers and practitioners, to better understand the model’s grounding in evidence-based practice.    

**Response**: Detailed information on PFA and MHFA in this context is provided in our previous feasibility study (Gorbunova et al., 2025). Relevant references are made in the text.

**Comment**: Furthermore, while the article rightly acknowledges the importance of supervision, quality assurance, and ongoing professional development, the discussion around monitoring and evaluation remains rather vague. A national rollout of this scale would benefit from a clear framework for assessing training quality, workforce retention of skills, and impact on referral pathways. The authors might consider outlining a basic monitoring and evaluation plan, even if in schematic form, identifying key indicators and potential data sources. This would give the proposal greater rigour and support future scalability and adaptation.         

**Response**: We have added a new subsection, 'Monitoring and Evaluation Framework for National Scaling'. This framework identifies specific indicators for training fidelity, workforce skill retention, the effectiveness of referral pathways, etc.

**Comment**: Equity is another area that warrants further attention. While the UMHT initiative is positioned as universal, there is little discussion of how it will reach or serve marginalized populations—such as rural residents, displaced persons, or minority groups. Given Ukraine’s socio-political context and the disparities in mental health access, the authors should consider briefly reflecting on how equity considerations will be embedded in the design, delivery, and evaluation of UMHT. This could include measures such as targeted outreach, linguistic or cultural adaptation, or disaggregated monitoring data.   

**Response**: We have added a new subsection, 'Embedding Equity and Inclusion', which outlines our strategy for reaching rural residents, IDPs, and minority groups.

**Comment**: Finally, a few stylistic and language issues are worth noting. The manuscript would benefit from minor editorial refinement, particularly in terms of phrasing and grammatical consistency. Terms like “stigma redaction” should be corrected to “stigma reduction”, and long paragraphs could be broken down for improved readability. Additionally, Figure 1, while helpful, could use a clearer caption and more legible formatting to ensure accessibility across platforms.

**Response**: Thank you, well noted

**Comment**: However, in order to be scientifically sound and persuasive for both policymakers and academic readers, the authors could strengthen the evidence base, clarify conceptual linkages to global models like PFA, and more explicitly outline strategies for evaluation and equity.

**Response**: Thank you, we believe that in the previous corrections we addressed all the mentioned issues.

# Reviewer report: Reviewer response for version 1

**DOI:** 10.21956/openreseurope.21760.r58204

**Published:** 2025-09-10

**Authors:** Joerg M Fegert, Emily Sitarski

**Affiliations:** 1 Department of Child and Adolescent Psychiatry, Psychosomatics and Psychotherapy, Universitatsklinikum Ulm, Ulm, Baden-Württemberg, Germany

Thank you very much for providing me with the essay “Universal mental health training: policy proposal for Ukraine.” The proposal recommends scaling up Universal Mental Health Training (UMHT) in Ukraine to equip frontline professionals, such as teachers, police, and social workers with basic skills to identify and respond to mental health needs. Aligned with Ukraine’s 2025 Mental Health Law, UMHT aims to close treatment gaps by expanding community-level support. Key challenges such as role integration, sustainable financing, and stigma reduction as well as the authors' proposed solutions are presented. The essay is well structured, clearly written, and deals with a socially and politically very important project that I strongly support. Nevertheless, it has some weaknesses that I would like to address. With regard to the integration of the current state of research and the supporting evidence for the written content, I have given a “Partly” rating, because in my opinion, the essay could be further enriched with literature in many places and is too brief in parts.

- First of all, I would like to point out that the title is hold very brief. It could be enriched with more information about the training or the proposal in general to make it more unambiguous and clear
-  Furthermore, the abstract mentions the new Ukrainian law on the Mental Health Care System, which is also mentioned later on, but it gives no explanation. More information on the content of the law and its implications would be helpful in order to better understand the topic
-  The introduction mentions a great need for psychological support among the Ukrainian population - is there any data on this? Furthermore, three consecutive Ukrainian initiatives are mentioned, but these are not explained - this would be helpful for better understanding
- The section on the background and pilot implementations of the health training mentions global projects such as the WHO's Mental Health Gap Action Programme and the Mental Health First Aid Programme, which form the basis for the current Ukrainian programme. However, the pioneers are not explained in any further detail, and it is not clear why these particular programmes were chosen – this would help to identify parallels and make the argument more fluid for the reader
- With regard to the challenges associated with implementing UMHT, I wondered whether these could be enriched with more data, perhaps even from Ukraine. This would make the argument for combating these challenges more convincing - for example, if it became clear exactly how much money is available for funding or what the general acceptance of psychological support services is like in Ukraine
- The section on “Public and Professional Attitudes” states that changes in people's mindsets are difficult - are there any figures on this? In my view, this section makes some claims for which it would be interesting seeing the evidence
- This impression also persists in the outlook for 2030 and the conclusion. Regarding the expected outcomes, I wondered whether it would be possible to give a look into initial pilot data. This would substantiate the assumptions, as this section currently reads very vaguely. And if this is not possible, is there evidence for other programs that have achieved these outcomes precisely?
- The conclusion reiterates why it is important to implement UMHT, as small gestures on the part of professionals can already make a big difference. Here, too, I wondered whether there is any concrete data that could be incorporated. For example, to what extent does it make a difference if police officers react with understanding rather than otherwise?
- Finally, the manuscript would benefit from proofreading and editing. For example, "Stigma redaction and rising awareness" should be written as "Stigma reduction and raising awareness," and "In 2023, 90 trainings will be conducted..." It should be written in the past tense.

Is the argument persuasive and supported by appropriate evidence?

Partly

Is the work clearly and cogently presented?

Yes

Is the topic of the essay discussed accurately in the context of the current literature?

Partly

Does the essay contribute to the cultural, historical, social understanding of the field?

Yes

Reviewer Expertise:

neglect, maltreatment, sexual abuse and other adverse childhood experiences as well as early intervention, the relationship between youth welfare and child and adolescent psychiatry and other legal and forensic issues, public child mental health

We confirm that we have read this submission and believe that we have an appropriate level of expertise to confirm that it is of an acceptable scientific standard, however we have significant reservations, as outlined above.

# Response

**Published:** 2025-12-26

**Authors:** Viktoriia Gorbunova

**Affiliations:** Department of Behavioural and Cognitive Sciences, University of Luxembourg, Esch-sur-Alzette, Luxembourg District, Luxembourg

We would like to thank the reviewers for their thoughtful and constructive feedback on our manuscript. Their insights have been invaluable in strengthening the empirical foundation and practical framework of this policy proposal. In response to the reviewers' comments, we have significantly revised the manuscript, addressing all the issues.

**Comment**: First of all, I would like to point out that the title is hold very brief. It could be enriched with more information about the training or the proposal in general to make it more unambiguous and clearer      

**Response**: We changed it to the “Universal mental health training: policy proposal for national rollout of the new scalable intervention in Ukraine”

**Comment**: Furthermore, the abstract mentions the new Ukrainian law on the Mental Health Care System, which is also mentioned later on, but it gives no explanation. More information on the content of the law and its implications would be helpful in order to better understand the topic         

**Response**: After each mentioning of the relevant documents, brief description of the implication is provided.

**Comment**: The introduction mentions a great need for psychological support among the Ukrainian population - is there any data on this? Furthermore, three consecutive Ukrainian initiatives are mentioned, but these are not explained - this would be helpful for a better understanding 

**Response**: Additional information on the prevalence of mental health problems is provided now, after the introductory sentences.

**Comment**: The section on the background and pilot implementations of the health training mentions global projects such as the WHO's Mental Health Gap Action Programme and the Mental Health First Aid Programme, which form the basis for the current Ukrainian programme. However, the pioneers are not explained in any further detail, and it is not clear why these particular programmes were chosen – this would help to identify parallels and make the argument more fluid for the reader

**Response**: Detailed information on PFA and MHFA in this context is provided in our previous feasibility study (Gorbunova et al., 2025). Relevant references are made in the text.

**Comment:** With regard to the challenges associated with implementing UMHT, I wondered whether these could be enriched with more data, perhaps even from Ukraine. This would make the argument for combating these challenges more convincing - for example, if it became clear exactly how much money is available for funding or what the general acceptance of psychological support services is like in Ukraine    

**Response**: Thank you, it would be very relevant indeed. However, no such data are available as of now.

**Comment**: The section on “Public and Professional Attitudes” states that changes in people's mindsets are difficult - are there any figures on this? In my view, this section makes some claims for which it would be interesting seeing the evidence   

**Response**: We deleted the general statement and left the rest. It is indeed very interesting to see the data; however, again, regarding the Ukrainian population, it is not available.

**Comment**: This impression also persists in the outlook for 2030 and the conclusion. Regarding the expected outcomes, I wondered whether it would be possible to give a look into initial pilot data. This would substantiate the assumptions, as this section currently reads very vaguely. And if this is not possible, is there evidence for other programs that have achieved these outcomes precisely?

**Response**: We amended the second sentence to add more clarity on the data: “Based on the data from the feasibility study and pilot trial (Gorbunova et al., 2024; Gorbunova et al., 2025), we can expect…”

**Comment**: The conclusion reiterates why it is important to implement UMHT, as small gestures on the part of professionals can already make a big difference. Here, too, I wondered whether there is any concrete data that could be incorporated. For example, to what extent does it make a difference if police officers react with understanding rather than otherwise?           

**Response**: We do hope that more data will be available in the near future.

**Comment**: Finally, the manuscript would benefit from proofreading and editing. For example, "Stigma redaction and rising awareness" should be written as "Stigma reduction and raising awareness," and "In 2023, 90 trainings will be conducted..." It should be written in the past tense.

**Response**: Thank you, well noted.

# Reviewer report: Reviewer response for version 1

**DOI:** 10.21956/openreseurope.21760.r58205

**Published:** 2025-09-04

**Authors:** Kathryn E Kanzler, Chase Aycock

**Affiliations:** 1 Department of Medicine; Department of Psychiatry & Behavioral Sciences, Baylor College of Medicine, Houston, Texas, USA; 2 Joint Base San Antonio - Wilford Hall Ambulatory Surgical Center, Lackland AFB, Texas, USA

We appreciated the opportunity to review the manuscript, “Universal mental health training: policy proposal for Ukraine.” This well-written and timely essay makes a compelling argument for scaling up Universal Mental Health Training (UMHT) in Ukraine. The challenges described appear significant, yet not insurmountable, given the solutions proposed by the authors – which were practical and likely to be useful. Additionally, Figure 1 is helpful in linking the challenges and recommendations together. We have generally minor feedback; we hope these recommendations will enhance this excellent essay.

**Introduction**

- Please rephrase this sentence in the first paragraph for clarity: “Mentioned low endorsements reconceptualize mental health care in Ukraine as a broad, community-based system in contrast to the old one restricted to psychiatric institutions.” 
- Consider adding “non-clinical” to describe social workers, to distinguish from those who are licensed clinical social workers: “an initiative to train and mobilize frontline professionals (such as teachers, police officers, non-clinical social workers, and others)”

**Universal Mental Health Training: background and pilot implementation results**

- In the first sentence, the author describes the “mental health treatment gap,” but it would be beneficial here to cite a study describing the extent of this problem related to this gap, providing relevant data. For instance, “XX% of individuals who need care do not receive it.” This addition would help to emphasize why there is such a pressing need for initiatives like UMHT.
- The last clause of this sentence needs to be rephrased to improve clarity. “Usability” is unclear. “The trainees reported increased knowledge and confidence in handling mental health-related work situations, and highlighted preparedness to use trained skills and their actual usability.”

**Challenges in the UMHT scaling up at the national level**

- Workforce quality assurance: This challenge has less explanation. It would be helpful to expand on why “quality control across a broad rollout” is challenging.
-  Public and Professional Attitudes:
  - The authors express realistic concern that the value of UMHT may be questioned by both frontline professionals and the general public. While they propose a response in the section titled “Mental Health Promotion and Stigma Reduction,” an additional persuasive strategy is found in the final paragraph of “Looking Ahead to 2030.” There, the authors cite data suggesting that frontline workers could experience increased job satisfaction and improved effectiveness through participating in UMHT. Highlighting these benefits could strengthen professional buy-in and positively shift attitudes. Therefore, we recommend incorporating this point into a new subsection under “Policy Actions to Overcome the Challenges and Scale Up the UMHT,” and reflecting it in Figure 1 under the category “Public and Professional Attitudes.” This change would also provide two solutions, aligning with the other identified challenges.
  - Please rephrase this sentence for clarity: “Both issues are related to stigma redaction and rising awareness as the only ways to overcome attitudinal barriers.”

**Policy Actions to Overcome the Challenges and Scale Up the UMHT**

- To enhance alignment with Figure 1, we recommend reordering the paragraph topics within this section so that it’s easier for the reader to follow how these proposed solutions relate to the challenges presented. This sequencing would improve the logical progression of the recommended solutions with the figure.

**Policy actions to overcome the challenges and scale up the UMHT**

- Sustainable financing strategy: Please verify that “continuous professional education” matches the acronym CPD. Should this be “Continuous Professional Development”? [Also, for scalability and sustainability, this is often addressed by Computer-Based Trainings (CBTs) in other contexts and could be recommended - although the effectiveness of those is not often measured or evaluated.]
- Mental Health Promotion and Stigma Reduction: Consider rephrasing the last part of the sentence, “To increase the buy-in of the UMHT by relevant local and national stakeholders, it is important to …”.  For example, edit to, “and integrate into them the idea that mental health support is everyone’s business.”

**Looking ahead to 2030 – Expected outcomes**

- It would strengthen your argument for UMHT to highlight that these improvements to the “mental health landscape” would occur without substantive changes to the mental health workforce (i.e., increased number of licensed MH providers).
- The authors highlight the expected outcomes well, in terms of improving access to care. However, it could be helpful toward the end of this article to again emphasize the pressing public health concern in terms of population-level burden for mental health concerns (e.g., “given the rising prevalence of XYZ in Ukraine…”) to emphasize the urgency of enacting scalable, universal mental health support.

The authors are to be commended again for outlining a clear and actionable path for Ukraine to broadly implement UMHT. The proposed rollout has the potential not only to address national mental health needs but also to serve as a model for other countries seeking to strengthen their population's mental health.

Is the argument persuasive and supported by appropriate evidence?

Yes

Is the work clearly and cogently presented?

Yes

Is the topic of the essay discussed accurately in the context of the current literature?

Partly

Does the essay contribute to the cultural, historical, social understanding of the field?

Yes

Reviewer Expertise:

task-shifting; implementation science; health equity; chronic disease management

We confirm that we have read this submission and believe that we have an appropriate level of expertise to confirm that it is of an acceptable scientific standard, however we have significant reservations, as outlined above.

# Response

**Published:** 2025-12-26

**Authors:** Viktoriia Gorbunova

**Affiliations:** Department of Behavioural and Cognitive Sciences, University of Luxembourg, Esch-sur-Alzette, Luxembourg District, Luxembourg

We would like to thank the reviewers for their thoughtful and constructive feedback on our manuscript. Their insights have been invaluable in strengthening the empirical foundation and practical framework of this policy proposal. In response to the reviewers' comments, we have significantly revised the manuscript, addressing all the issues.

**Comment:** Please rephrase this sentence in the first paragraph for clarity: “Mentioned low endorsements reconceptualize mental health care in Ukraine as a broad, community-based system in contrast to the old one restricted to psychiatric institutions.”

**Response: **“Mentioned legislative endorsements reconceptualize mental health care in Ukraine as a broad, community-based system in contrast to the old one restricted to psychiatric institutions”

**Comment:** Consider adding “non-clinical” to describe social workers, to distinguish from those who are licensed clinical social workers: “an initiative to train and mobilize frontline professionals (such as teachers, police officers, non-clinical social workers, and others)” **Response:** Word “non-clinical” is added for the clarity

**Comment: **In the first sentence, the author describes the “mental health treatment gap,” but it would be beneficial here to cite a study describing the extent of this problem related to this gap, providing relevant data. For instance, “XX% of individuals who need care do not receive it.” This addition would help to emphasize why there is such a pressing need for initiatives like UMHT.          

**Response:** Unfortunately, such data are not available for Ukraine; therefore, it is impossible to make relevant references to a reliable source.

**Comment:** The last clause of this sentence needs to be rephrased to improve clarity. “Usability” is unclear. “The trainees reported increased knowledge and confidence in handling mental health-related work situations, and highlighted preparedness to use trained skills and their actual usability.”        

**Response:** The pointed sentence is corrected: «The trainees reported increased knowledge and confidence in handling mental health-related work situations, and highlighted preparedness to use skills received during training»

**Comment**: Workforce quality assurance: This challenge has less explanation. It would be helpful to expand on why “quality control across a broad rollout” is challenging.

**Response:** To add clarity, the second sentence in the paragraph was extended: “Maintaining trainees’ engagement and updating their skills over time requires ongoing supervision and upskilling, which in turn requires a substantial increase in trained professionals and the integration of UMHT into the system of continuous professional development across different sectors (which are usually non-integrated).”

**Comment**: Public and Professional Attitudes: The authors express realistic concern that the value of UMHT may be questioned by both frontline professionals and the general public. While they propose a response in the section titled “Mental Health Promotion and Stigma Reduction,” an additional persuasive strategy is found in the final paragraph of “Looking Ahead to 2030.” There, the authors cite data suggesting that frontline workers could experience increased job satisfaction and improved effectiveness through participating in UMHT. Highlighting these benefits could strengthen professional buy-in and positively shift attitudes. Therefore, we recommend incorporating this point into a new subsection under “Policy Actions to Overcome the Challenges and Scale Up the UMHT,” and reflecting it in Figure 1 under the category “Public and Professional Attitudes.” This change would also provide two solutions, aligning with the other identified challenges.  

**Response:** Thank you, well noted! The mentioned part from “Looking Ahead to 2030.” Was edited and moved to the “Mental Health Promotion and Stigma Reduction” section: “The frontline workers themselves may actually find their work easier in some respects – many report that after UMHT training, they felt better prepared when encountering mental health issues. Empowerment can lead to greater job satisfaction and greater effectiveness, and less stigma.”  However, we decided against making the second sub-strategy, as this phenomenon is merely the observation, and not something that can be promoted under controllable conditions.

**Comment:** Please rephrase this sentence for clarity: “Both issues are related to stigma redaction and rising awareness as the only ways to overcome attitudinal barriers.” **Response:** Thank you, sentence is edited: “Both issues are related to stigma reduction, and raising awareness may help overcome attitudinal barriers”

**Comment**: Policy Actions to Overcome the Challenges and Scale Up the UMHT. To enhance alignment with Figure 1, we recommend reordering the paragraph topics within this section so that it’s easier for the reader to follow how these proposed solutions relate to the challenges presented. This sequencing would improve the logical progression of the recommended solutions with the figure.      

**Response**: Thank you, we changed the sequence – it is now correlated to the Figure 1.

**Comment**: Sustainable financing strategy: Please verify that “continuous professional education” matches the acronym CPD. Should this be “Continuous Professional Development”? [Also, for scalability and sustainability, this is often addressed by Computer-Based Trainings (CBTs) in other contexts and could be recommended - although the effectiveness of those is not often measured or evaluated.]    

**Response**: Yes, it has to be “Continuous Professional Development” – corrected

**Comment**: Mental Health Promotion and Stigma Reduction: Consider rephrasing the last part of the sentence, “To increase the buy-in of the UMHT by relevant local and national stakeholders, it is important to …”.  For example, edit to, “and integrate into them the idea that mental health support is everyone’s business.” 

**Response**: Relevant correction was made

**Comment**: Looking ahead to 2030 – Expected outcomes. It would strengthen your argument for UMHT to highlight that these improvements to the “mental health landscape” would occur without substantive changes to the mental health workforce (i.e., an increased number of licensed MH providers).      

**Response**: Great suggestion, implemented: “It is important to note that such changes would occur without substantive increases in the number of mental health workers, and therefore with minimal financial investment.”

**Comment**: The authors highlight the expected outcomes well, in terms of improving access to care. However, it could be helpful toward the end of this article to again emphasise the pressing public health concern in terms of the population-level burden of mental health conditions (e.g., “given the rising prevalence of XYZ in Ukraine…”) to underscore the urgency of enacting scalable, universal mental health support.  

**Response**: At the end of the Conclusion, such a sentence was added: “Given the rapidly rising prevalence of mental health conditions across Ukraine, the urgency of implementing scalable mental health solutions becomes even more evident, underscoring the public health imperative at a population level.”
